# Betulinic Acid Reduces Intestinal Inflammation and Enhances Intestinal Tight Junctions by Modulating the PPAR-γ/NF-κB Signaling Pathway in Intestinal Cells and Organoids

**DOI:** 10.3390/nu17132052

**Published:** 2025-06-20

**Authors:** Xu Zheng, Zhen Cao, Mingqi Wang, Ruqiang Yuan, Yinhe Han, Ang Li, Xiuli Wang

**Affiliations:** 1 College of Basic Medical Science, Dalian Medical University, Dalian 116044, China; zhengxu@dmu.edu.cn (X.Z.); caozhen@stu.glmc.edu.cn (Z.C.); wangmq@dmu.edu.cn (M.W.); yuanrq666@163.com (R.Y.); han1993yh@163.com (Y.H.); drang33@outlook.com (A.L.); 2 Advanced Institute for Medical Sciences, Dalian Medical University, Dalian 116044, China

**Keywords:** betulinic acid, inflammatory bowel disease, intestinal organoids, intestinal epithelial barrier, PPAR-γ

## Abstract

**Background:** Intestinal epithelial barrier (IEB) dysfunction is related to multiple gastrointestinal disorders, notably inflammatory bowel disease (IBD). Betulinic acid (BA), a compound derived from birch bark, has demonstrated potential therapeutic benefits in IBD. Nevertheless, the impact of BA on IEB function has not been fully elucidated. **Methods:** The current study aimed to explore the potential underlying mechanisms of BA in dextran sodium sulfate (DSS)-induced IBD in mice and co-culture models involving Caco-2/HT29-MTX-E12 cell monolayers or mouse intestinal organoids (IOs) in conjunction with macrophages stimulated by lipopolysaccharide (LPS). **Results:** In vivo, BA treatment significantly improved body weight and colon length, alleviated disease activity index (DAI) scores, and reduced colonic histopathological injury in IBD mice. In vitro, BA reduced the flux of FITC-dextran; increased the TEER; and decreased the production of IL-6, IL-1β, and TNF-α while increasing IL-10 mRNA levels. Additionally, BA enhanced IEB formation by upregulating ZO-1, occludin (OCLN), and claudin-1 (CLDN1). Molecular docking studies revealed significant docking scores and interactions between BA and PPAR-γ. Moreover, BA significantly upregulated PPAR-γ protein expression, decreased NF-κB and MLC2 phosphorylation, and reduced MLCK protein expression. However, this effect was reversed by GW9662, an effective PPAR-γ antagonist. **Conclusions:** The findings reveal that BA mitigates IBD by safeguarding the intestinal barrier against dysfunction. This effect may be attributed to its ability to suppress inflammation and enhance the expression of tight junction proteins by modulating the PPAR-γ/NF-κB signaling pathway.

## 1. Introduction

The intestinal epithelial barrier (IEB) plays pivotal roles in safeguarding the structural integrity of the gut lining, preventing pathogenic infiltration, and regulating intestinal homeostasis [1]. Studies have demonstrated that IEB dysfunction is linked to a wide spectrum of pathologies, encompassing gastrointestinal diseases, neurological disorders, allergic reactions, and metabolic dysfunction [2,3,4,5]. The protective barrier function of intestinal epithelial cells (IECs), which serves as the primary interface separating the gut lumen from the lamina propria, is predominantly regulated by tight junction (TJ) complexes [6]. The pathogenesis of inflammatory bowel disease (IBD) involves intestinal barrier dysfunction, which facilitates the translocation of luminal antigens across the epithelial layer. This breach initiates immune activation and the subsequent production of pro-inflammatory cytokines [7]. Thus, approaches focused on enhancing the intestinal TJ barrier might be essential in inhibiting the development and progression of intestinal inflammation.

Betulinic acid (BA), a pentacyclic triterpenoid compound, has emerged as a lead candidate for anti-inflammatory drug development, with numerous structural analogs currently under investigation for their pharmacological properties [8]. Preclinical investigations across multiple experimental models have demonstrated reproducible anti-inflammatory effects of BA and its structural derivatives [9]. Recent studies have demonstrated that BA exhibits immunomodulatory properties through its ability to regulate diverse cellular populations and signaling pathways implicated in inflammatory cascades [10,11,12]. Notably, in vitro evidence has indicated that BA suppresses lipopolysaccharide (LPS)-induced nitric oxide (NO) synthesis, particularly in activated macrophage populations [13]. Experimental studies in murine colitis models have demonstrated that BA significantly attenuates myeloperoxidase (MPO) activity and lipid peroxide levels in colonic tissue while restoring catalase and superoxide dismutase (SOD) antioxidant functions. Furthermore, BA treatment reduces glutathione depletion, suggesting a protective role against oxidative stress. Consequently, these effects lead to a significant downregulation of matrix metalloproteinase-9 (MMP-9) and prostaglandin E2 (PGE2) expression [14]. Despite these promising findings, the precise mechanisms through which BA confers protection to intestinal IECs during inflammation remain incompletely elucidated and require further investigation.

The present study aimed to explore the impact of BA on IEB functionality both in vivo and in vitro. BA was administered to DSS-induced murine models of IBD. Additionally, an in vitro inflammatory model was established using a co-culture system of Caco-2 and HT29-MTX-E12 cell lines, which are human-colorectal-adenocarcinoma-derived cell lines modeling intestinal epithelial and mucus-secreting cells, respectively [15]. To recapitulate IBD-associated inflammation, a Transwell co-culture system combining epithelial cells (Caco-2/HT29-MTX-E12) with RAW264.7 macrophages was established. The system was stimulated with LPS to induce a robust pro-inflammatory response, mimicking the immune–epithelial crosstalk characteristic of IBD pathophysiology [16]. Acknowledging the limitations of cell lines in modeling the full complexity of IECs, our studies were complemented with mouse intestinal organoids (IOs) co-cultured with RAW264.7 macrophages. This integrated model better recapitulates intestinal cellular diversity and enables a more comprehensive investigation of barrier function and immune–epithelial interactions, thereby facilitating the development of novel IBD therapeutics.

## 2. Materials and Methods

### 2.1. Materials and Reagents

Betulinic acid (HY-10529) and GW9662 (HY-10108) were sourced from MedChemExpress (Shanghai, China). LPS was obtained from Sigma (St. Louis, MO, USA), and FITC-dextran 4 was obtained from Maokangbio (Shanghai, China). DSS (D808272) was obtained from Macklin (Shanghai, China). EDTA, PBS, high-glucose DMEM, and penicillin–streptomycin were obtained from HyClone (Logan, UT, USA). Antibodies targeting PPAR-γ, MLCK, MLC2, pMLC2, NF-κB, pNF-κB, ZO-1, OCLN, CLDN1, β-actin, and GAPDH were acquired from Proteintech (Wuhan, China), Abcam (Cambridge, UK), Affinity Biosciences (Suzhou, China), HuaBio (Hangzhou, China), Cohesion (Suzhou, China), and ABclonal Technology (Wuhan, China), respectively.

### 2.2. Animal Experiments

**Figure 1:**
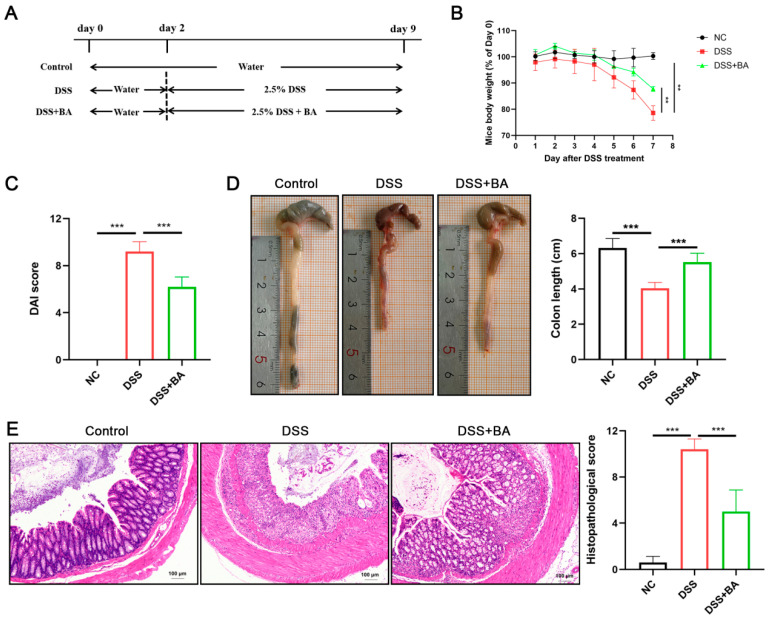
BA ameliorates DSS-induced IBD symptoms in mice. (**A**) Schematic overview of study design in the animal model. (**B**) Body weight change in mice after DSS treatment. (**C**) DAI score. (**D**) Representative images of mouse colon and colon length. (**E**) Representative HE-stained histological sections and histopathology scores. *n* = 5. ** *p* < 0.01, *** *p* < 0.001.

Eight-week-old male C57BL/6J mice (25 ± 1 g) obtained from Changsheng Biotechnology Co., Ltd. (Benxi, China), were used in this study. All experimental procedures were conducted in compliance with animal welfare guidelines and approved by the Animal Ethics Committee of Dalian Medical University (Approval No. AEE23106). A total of 15 mice were chosen according to the Sample Size Calculation for Animal Studies Using Degrees of Freedom (E). The animals were housed in polycarbonate cages with hardwood chips, where the temperature (23 ± 1 °C), lighting conditions (12 h light/dark cycle), and humidity (40–60%) were strictly controlled. The animals had free access to a standard diet and water ad libitum. After adapting for a week, the mice were randomly divided into three groups (*n* = 5 per group): a control group, which received standard drinking water for 9 days; a DSS group, which received drinking water for 2 days, followed by the administration of 2.5% DSS (*w*/*v*) in drinking water for 7 days; and a DSS + BA group, which was treated identically to the DSS group but with additional daily oral administration of BA (30 mg/kg [14]), as shown in Figure 1A. The DAI was evaluated on day 9 using a previously described method [17]. Following euthanasia via CO_2_ inhalation, colons were excised, measured, and processed for H&E staining. Histopathological scoring (0–4) was evaluated based on epithelial surface erosion, crypt architecture distortion, and immune cell infiltration [18].

### 2.3. Cell Culture

The cell lines Caco-2 (ATCC, HTB-37), HT29-MTX-E12 (ECACC, 12040401), and RAW264.7 (Pricella, Wuhan, China, CL-0190), which model intestinal epithelial cells, mucin-secreting goblet cells, and macrophages, respectively, were maintained individually in complete medium and subcultured every two days.

### 2.4. Cell Viability Assay

The following cell types were used: Caco-2 (3 × 10^3^ cells/well), HT29-MTX-E12 (4 × 10^3^ cells/well), and RAW264.7 (6 × 10^3^ cells/well). Inoculation was performed for 24 h after the cells were seeded into 96-well plates. Cell viability was assessed after 24 h using a CCK-8 kit (Dojindo, Kumamoto, Japan) according to protocols, following treatment with either DMSO (control) or different BA doses (5, 10, 20, 40, 80, and 160 µM). A multifunctional microplate reader (Thermo, Waltham, MA, USA) was used to detect absorbance at 450 nm. The proportion of live cells relative to that of the control was used to express viability.

### 2.5. Establishment of an Inflamed Epithelium–Microphage Co-Culture Model

The apical surface of cell culture inserts with 0.4 μm pores was seeded with 10^6^ cells/mL of Caco-2 and HT29-MTX-E12 cells at a 3:1 ratio, as previously mentioned [19]. On the fourteenth day after seeding, monolayers were used, with the culture medium changed every two to three days. RAW264.7 cells (10^6^ cells/mL per well) were seeded on day 13. On day 14, the control group contained growth medium on both sides without LPS or BA treatment, whereas the LPS group contained macrophages treated with LPS (1 µg/mL) in the basolateral section and only growth medium in the apical section. In the LPS + BA group, the cells were treated with BA (10 µM) on the apical side and LPS (1 µg/mL) on the basolateral side; in the LPS + BA + GW9662 group, the cells were treated with GW9662 (10 µM) and BA (10 µM) on the apical side, along with LPS on the basolateral side. After 24 h, the TEER value was measured using a Millicell-ERS2 Volt Ohmmeter (Millipore, Burlington, MA, USA), and it is expressed as a percentage of the control. After being transferred to a new plate, the apical monolayers were used for paracellular permeability assays and protein or mRNA detection.

### 2.6. Paracellular Permeability Assay

The use of FITC-dextran 4 allowed for an evaluation of paracellular permeability. After the monolayers had been gently rinsed with PBS, 1 mg/mL of FITC-dextran 4 was added to the apical compartment, and the basolateral chamber’s media were replaced with PBS. After incubation for 6 h, 100 µL of basolateral PBS was transferred to a black opaque 96-well plate. Next, the fluorescence intensity was measured using a multi-mode microplate reader at an excitation/emission wavelength of 493/520 nm. A fluorescence value of 100% was considered to have been achieved in the control cells.

### 2.7. IO Culture

Here, IOs were isolated and cultured from the small intestines of 6-week-old C57BL/6 mice following Wang et al.’s method [20]. Mouse intestines were sectioned, and intestinal crypts were isolated using a gentle cell dissociation reagent (Stemcell, Vancouver, BC, Canada). The digestion process was terminated by adding PBS containing 0.1% BSA. The crypt extract was then subjected to filtration through a 70 µm cell strainer, followed by centrifugation at 290× *g* for 5 min, and the resulting pellets were suspended in Matrigel for culture. A 30 µL droplet was deposited on a pre-heated 12-well plate to solidify for 30 min, after which 800 µL of complete medium was added. The IOs were passaged using both digestion and mechanical disruption.

### 2.8. Establishment of an Inflamed Mice IO–Microphage Co-Culture Model

Mouse IOs were seeded on the apical surface of a 24-well cell culture insert with dimensions of 0.3 cm^2^ and a pore size of 0.4 µm (Corning, Corning, NY, USA) for a period of one day. RAW264.7 cells were distributed into 24-well plates (10⁶ cells/mL per well) and allowed to settle overnight. After undergoing various treatments for two days, as outlined in Section 2.5, the IOs were photographed using a Leica TCS microscope (Leica, Wetzlar, Germany) and then immediately obtained for paracellular permeability evaluation, immunofluorescence detection, or mRNA extraction.

### 2.9. Measurement of TJ Permeability in Mouse IOs

To assess the relative permeability of the IOs, FITC-dextran 4 was introduced into the growth media, and the flux of FITC-dextran 4 into IOs with a mature columnar epithelium was measured after 8 h. Using a confocal laser scanning microscope (Leica, Germany) and following established techniques, a number of optical sections were acquired throughout each IO in order to quantify the fluorescence intensity in the middle of the lumen. The fluorescence intensity in the control cells was set to 100%.

### 2.10. Immunofluorescence

The IO mixture was rinsed once with PBS, then exposed to fixation overnight in 4% paraformaldehyde and embedded in paraffin. After preparing the samples, the slides and monolayers were treated with 0.5% Triton X-100 for 10 min, followed by a PBS rinse and a 30 min treatment with 3% BSA. The sections were subjected to overnight incubation at 4 °C with primary antibodies such as CLDN1, ZO-1, and OCLN rabbit pAbs (1:200). The samples were then exposed to 15 min of incubation at room temperature with goat anti-rabbit Alexa Fluor 488 or 549 (1:200) and DAPI (1:500; Beyotime, Shanghai, China), and they were examined using a Pannoramic MIDI (3DHISTECH, Budapest, Hungary).

### 2.11. Quantitative Real-Time PCR (qRT-PCR)

The samples were treated with TRIzol reagent (Ambion Life Technologies, Roskilde, Denmark) according to protocols in order to extract total RNA, and then 1 µg of RNA was reverse-transcribed into cDNA using a TaKaRa (Shiga, Japan) kit. An SYBR^®^ Premix Ex Taq™ II Kit (Takara Bio, Shiga, Japan) was used to perform qRT-PCR on an Agilent Technologies System (Beijing, China). The amplification cycle comprised 40 cycles of 3 s at 95 °C, 30 s at 58 °C, and 30 s at 72 °C. The 2^−ΔΔCt^ method, normalized to GAPDH, was used to determine gene expression, and duplicate samples were analyzed. The primer sequences are listed in Table S1.

### 2.12. Western Blot Analysis

An ice-cold lysis solution containing RIPA was used to disrupt the monolayers of differentiated HT29-MTX-E12 and Caco-2 cells. A BCA kit (Beyotime, China) was then employed to assess protein levels. The samples were loaded and separated using SDS-PAGE, with a total protein concentration of 20 µg, and then they were transferred to PVDF membranes. The membranes were blocked for 2 h at room temperature with 3% BSA. The membranes were then immunoblotted with primary antibodies specific to ZO-1, OCLN, CLDN1, PPAR-γ, NF-κB, pNF-κB, MLCK, MLC2, pMLC2 (1:1000), β-actin, and GAPDH (1:5000). The membranes were incubated with HRP-conjugated secondary antibody (1:4000) at room temperature for 1 h. Afterward, bands were visualized using enhanced ECL and quantified using ImageJ software (Fiji 1.54f).

### 2.13. Network Pharmacology Analysis

The target proteins of BA were defined by accessing the UniProt database (https://www.uniprot.org/) and mapping them to gene names. These targets were then imported into Cytoscape 3.9.1 to create a network. Using GeneCards (https://www.genecards.org/), OMIM (https://omim.org/), DrugBank (https://www.drugbank.ca/), and the Therapeutic Target Database (https://db.idrblab.net/ttd/), IBD-associated targets were identified. A Venn diagram was generated to highlight the overlap between BA and IBD-related genes.

The STRING 11.5 platform (https://string-db.org/) was used to upload the intersecting genes in order to determine the relationships of protein–protein interactions (PPIs). Except for adjusting the minimal interaction threshold to “medium confidence” at 0.7 and setting the species to “Homo sapiens” (human), all other settings remained at their default values.

A KEGG pathway analysis was performed to elucidate the roles of the intersecting genes of BA in signaling pathways. The data were sourced from the Metascape database (https://metascape.org/gp/index.html#/main/step1, accessed on 24 January 2024).

### 2.14. Molecular Modeling

The interaction between PPAR-γ and BA was analyzed through molecular docking (MOD) using AutoDock Vina 1.2.5, where the binding affinity (kcal/mol) reflects the binding free energy. Greater stability in ligand–receptor binding is associated with a more negative binding free energy value.

### 2.15. Statistical Analysis

For statistical analyses, we utilized GraphPad Prism 9 (GraphPad, San Diego, CA, USA) and performed a one-way ANOVA to compare several parameters. Data are presented as the mean ± standard deviation. A *p*-value of less than 0.05 was considered statistically significant following the necessary tests.

## 3. Results

### 3.1. BA Ameliorates DSS-Induced IBD Symptoms in Mice

An animal model of DSS-induced IBD in mice was established, as shown in Figure 1A. Compared to the controls, the DSS-treated mice exhibited markedly increased DAI scores and body weight reduction. In contrast, BA administration significantly lowered DAI scores and promoted weight recovery (Figure 1B,C). DSS treatment significantly shortened colon length compared to the control, whereas BA administration partially restored this DSS-induced reduction (Figure 1D). BA treatment significantly alleviated the infiltration of inflammatory cells and reduced the histopathological score of colonic tissues (Figure 1E). In summary, BA alleviated the DSS-induced IBD symptoms in the mice.

### 3.2. Assessment of BA’s Effects on IEC and Macrophage Viability

**Figure 2:**
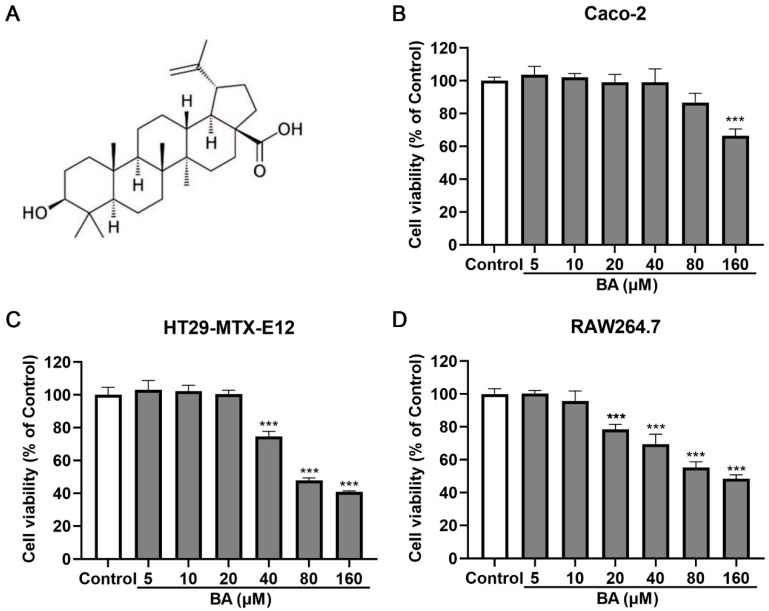
Cytotoxicity of BA on IEC and macrophage viability. (**A**) BA chemical structure. (**B**–**D**) Caco-2, HT29-MTX-E12, and RAW264.7 cells were treated with BA (0–160 μM). Cell viability was assessed using CCK-8 assay at 24 h. *n* = 3, *** *p* < 0.001.

As a pentacyclic triterpenoid (Figure 2A), BA has demonstrated selective cytotoxicity against various tumor cells, bacterial pathogens, and inflammatory processes. To assess its safety profile for intestinal applications, we first evaluated BA’s effects on IEC and macrophage viability using CCK-8 assays (Figure 2B–D). The results revealed no significant cytotoxicity in Caco-2 (0–80 µM), HT29-MTX-E12 (0–20 µM), or RAW264.7 cells (0–10 µM). Based on these findings, we selected 10 µM BA for subsequent experiments, as this concentration was non-toxic across all cell types while remaining within the biologically active range reported in the literature.

### 3.3. BA Restores IEB Integrity, Decreases Inflammation, and Enhances TJ Protein Expression in the Cell Monolayer

**Figure 3:**
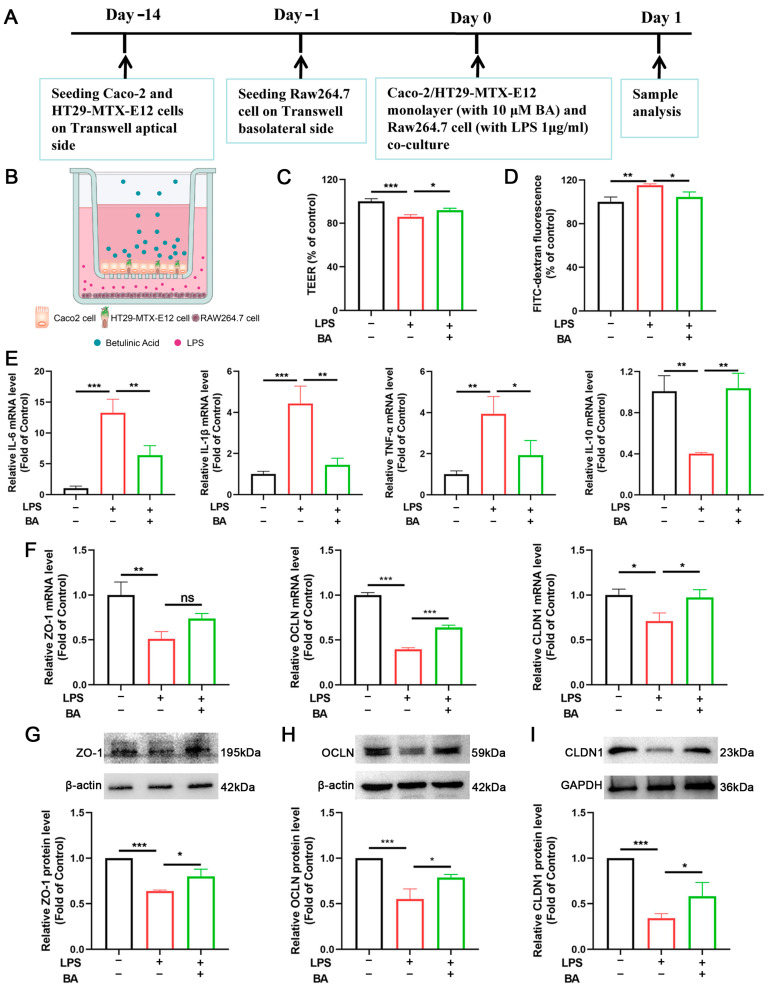
BA restores IEB integrity, decreases inflammation, and enhances TJ protein expression in the cell monolayer. (**A**) Schematic overview of study design in cell monolayer model. (**B**) Co-culture configuration: Caco-2/HT29-MTX-E12 on the apical side, RAW264.7 on the basolateral side. (**C**) Transepithelial electrical resistance (TEER). (**D**) Paracellular flux (FITC-dextran). (**E**) IL-6, IL-1β, IL-10, and TNF-α mRNA expression. (**F**) ZO-1, OCLN, and CLDN1 mRNA expression. (**G**–**I**) ZO-1, OCLN, and CLDN1 protein bands and relative expression. *n* = 3, * *p* < 0.05, ** *p* < 0.01, *** *p* < 0.001, ns: not significant.

A central objective of this study was to establish an in vitro human intestinal model that accurately recapitulates both the physiological barrier properties and inflammatory responses characteristic of IBD. The model comprised a polarized epithelial monolayer established by co-culturing Caco-2 and HT29-MTX-E12 cells at a 3:1 ratio (apical side) for 14 days. On day 13, RAW264.7 macrophages were seeded on the basolateral side and allowed to adhere overnight. Subsequently, the apical compartment was treated with 10 µM BA, while the basolateral side was challenged with 1 mg/mL LPS for 24 h (Figure 3A,B). LPS stimulation markedly reduced TEER while increasing paracellular permeability, as evidenced by elevated FITC-dextran flux (Figure 3C,D), which collectively indicates compromised intestinal barrier integrity. LPS stimulation significantly upregulated the mRNA expression of pro-inflammatory cytokines (IL-6, IL-1β, and TNF-α) while downregulating the anti-inflammatory cytokine IL-10. Notably, BA treatment effectively reversed these inflammatory mediators to near-baseline levels (Figure 3E).

Prolonged exposure to pro-inflammatory mediators disrupts TJ proteins, leading to increased intestinal permeability. As shown in Figure 3F, a qRT-PCR analysis revealed that the LPS group had significantly lower mRNA levels of ZO-1, OCLN, and CLDN1 than the control. Notably, BA treatment reversed these effects, restoring the mRNA expression of these crucial TJ proteins. Western blotting results further supported the protective role of BA by demonstrating the restoration of ZO-1, OCLN, and CLDN1 protein expression (Figure 3G–I). These results suggest that BA contributes to maintaining the integrity of the intestinal epithelial barrier (IEB) by reducing inflammation and enhancing tight junction function.

### 3.4. BA Reduces Inflammation and Enhances Tight Junction Integrity in the IOs–Macrophage Co-Culture System

**Figure 4:**
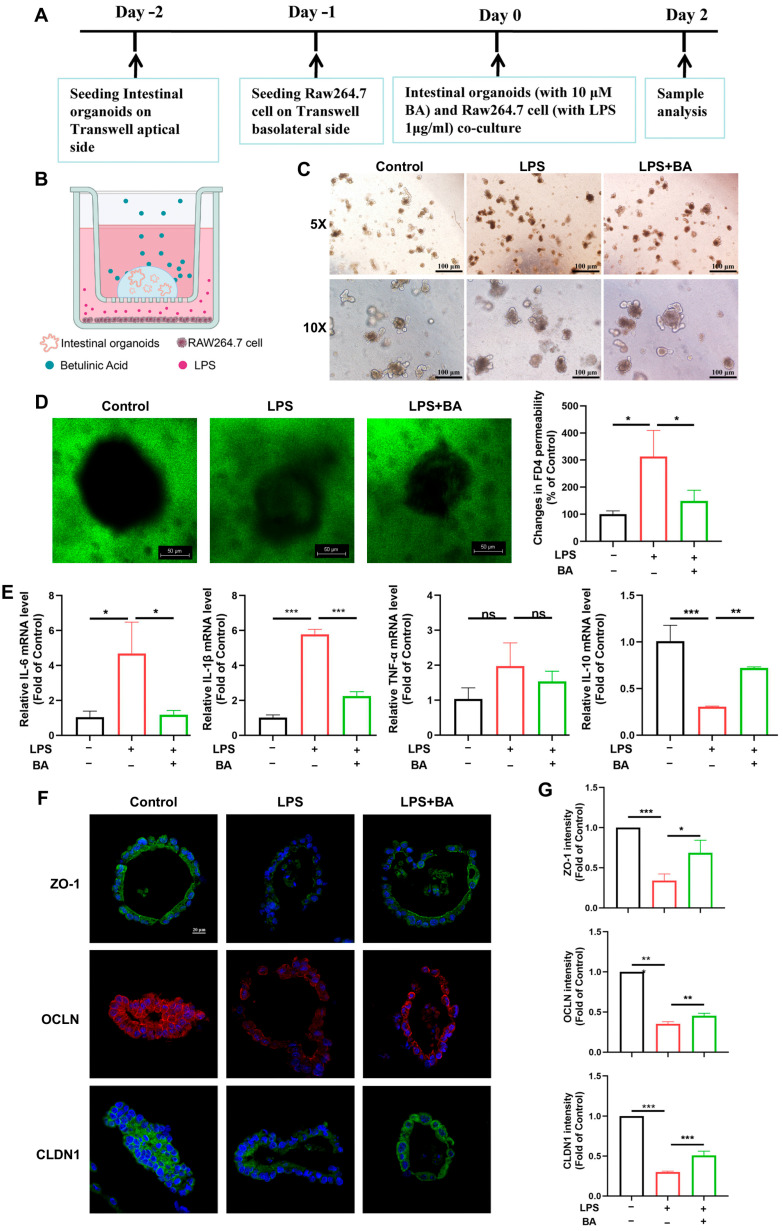
BA reduces inflammation and enhances tight junction integrity in the IEC–macrophage co-culture system. (**A**) Schematic overview of study design in IO model. (**B**) Schematic overview of the IO grown on the cell Transwell apical side. Activated RAW264.7 cells are on the basolateral side. (**C**) Morphology of IOs. (**D**) FITC-dextran fluorescence value in IOs-RAW264.7 co-culture model. (**E**) IL-6, IL-1β, IL-10, and TNF-α mRNA expression. (**F**,**G**) Immunofluorescence was used to show the distribution and expression of ZO-1, OCLN, and CLDN1 in IOs. ns, not significant, *n* = 3, * *p* < 0.05, ** *p* < 0.01, *** *p* < 0.001, ns: not significant.

To better recapitulate the physiological complexity of the IEB, we established an advanced in vitro co-culture system combining mouse IOs with RAW264.7 macrophages. This model allowed for a mechanistic investigation of BA’s protective effects on IEB integrity during inflammatory challenges. IOs were passaged and seeded apically, followed by the basolateral addition of RAW264.7 macrophages after 24 h. The system was then treated with 10 µM BA (apical) while inducing inflammation via 1 mg/mL LPS (basolateral) for 24 h (Figure 4A,B). LPS reduced decreased organoid budding activity. Notably, BA treatment significantly attenuated these changes, restoring near-normal organoid architecture (Figure 4C). LPS exposure significantly increased intestinal permeability (as measured by FITC-dextran flux) and elevated the expression of pro-inflammatory cytokines (IL-6, IL-1β, and TNF-α) in IOs, confirming epithelial barrier dysfunction. However, BA treatment restored barrier integrity by reducing permeability and IL-6 and IL-1β levels while upregulating the anti-inflammatory cytokine IL-10, although it did not significantly alter TNF-α production (Figure 4D,E). An immunofluorescence analysis of TJ proteins was conducted to evaluate epithelial barrier integrity in IOs. The IOs in the control group exhibited characteristic apical junctional localization of these proteins, consistent with intact barrier function. However, co-culture with LPS-activated RAW264.7 macrophages induced significant TJ disruption, as evidenced by a diminished expression and aberrant redistribution of ZO-1, OCLN, and CLDN1 from cell–cell contact sites.

BA treatment effectively restored the physiological localization of TJ proteins (Figure 4F,G), demonstrating its protective role in preserving epithelial barrier integrity under inflammatory conditions.

### 3.5. BA Engages with PPAR-γ Signaling Pathway Based on Network Pharmacology and MOD

**Figure 5:**
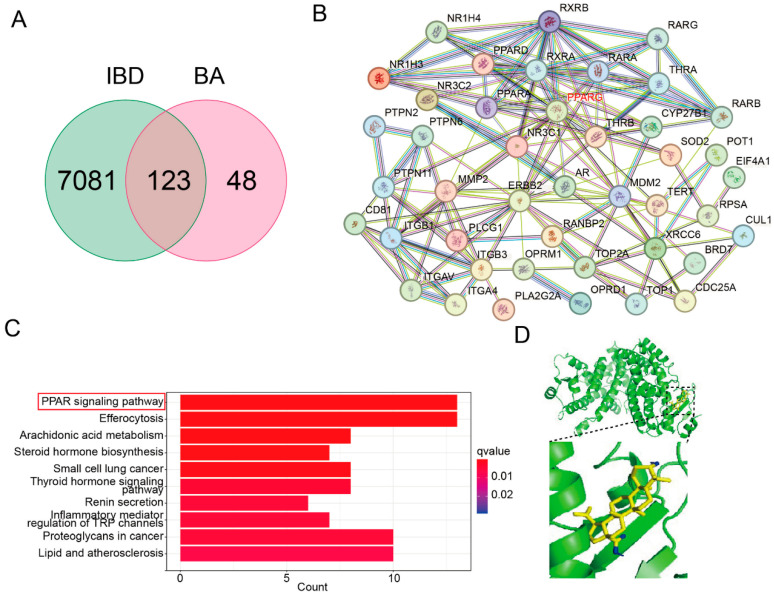
BA engages with PPAR-γ signaling pathway based on network pharmacology and MOD. (**A**) Venn diagram. (**B**) PPI network. (**C**) KEGG. (**D**) MOD.

To elucidate the mechanisms underlying BA’s protective effects on IEB dysfunction, we employed network pharmacology and MOD analyses. Our integrated approach identified 123 potential BA-targeted proteins interacting with 7204 IBD-associated genes, revealing a comprehensive therapeutic network (Figure 5A). Network visualization and topological analyses were performed using Cytoscape (v3.8.2), generating a protein–protein interaction network (Figure 5B) and its associated topological parameters. A KEGG pathway enrichment analysis further revealed that BA’s protective effects on inflammation-induced IEB dysfunction may be mediated by the regulation of the PPAR signaling pathway (Figure 5C). A molecular docking analysis revealed strong binding between BA and PPAR-γ (a docking score of −8.4 kcal/mol) (Figure 5D), demonstrating direct interaction with this key immunomodulatory receptor. This finding supports BA’s therapeutic potential in maintaining intestinal barrier integrity through the PPAR-γ-mediated regulation of inflammatory pathways.

### 3.6. BA Repairs IEB Dysfunction in the IEC Monolayer by Modulating the PPAR-γ/NF-κB Signaling Pathway

**Figure 6:**
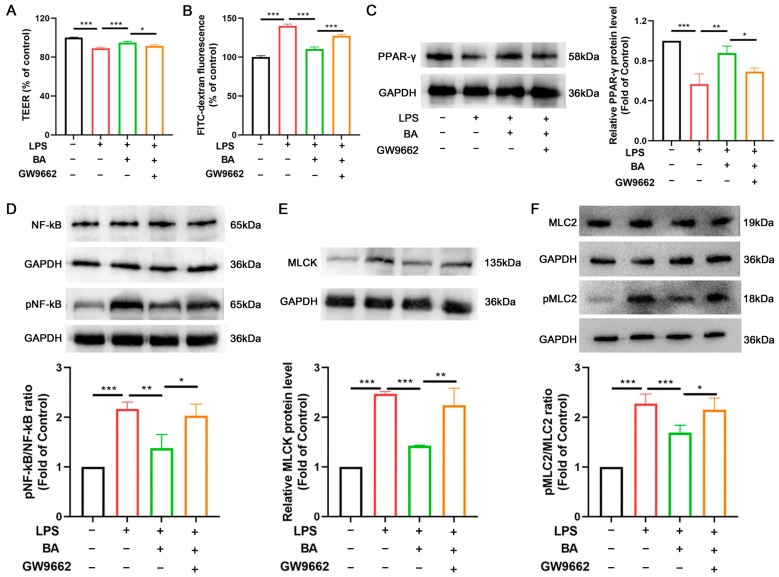
BA repairs IEB dysfunction in the IEC monolayer by modulating the PPAR-γ/NF-κB signaling pathway. (**A**) Transepithelial electrical resistance (TEER). (**B**) Paracellular flux (FITC-dextran). (**C**) PPAR-γ protein expression. (**D**–**F**) pNF-κB, NF-κB, MLCK, pMLC2, and MLC2 protein expression. *n* = 3, * *p* < 0.05, ** *p* < 0.01, *** *p* < 0.001.

To confirm the effects of BA on the PPAR-γ signaling pathway, we applied the PPAR-γ antagonist GW9662 in the Caco-2/HT29-MTX-E12-macrophage co-culture system. Compared to LPS treatment, BA led to a notable increase in TEER and a decrease in FITC-dextran permeability, although these effects were negated by the PPAR-γ antagonist GW9662 in the IEC monolayer (Figure 6A,B). PPAR-γ can be inhibited by LPS and pro-inflammatory cytokines, which activate NF-κB signaling. The NF-κB/MLCK pathway has been found to be essential for governing intestinal barrier integrity under pathophysiological conditions. The present study revealed that PPAR-γ expression decreased while pNF-κB/NF-κB, MLCK, and pMLC2/MLC2 protein levels increased in the LPS group. BA reversed these protein expression changes; however, GW9662 treatment inhibited BA’s effects on the IEC monolayer (Figure 6C–F). According to these results, BA repairs LPS-induced IEB damage by activating PPAR-γ and inhibiting the NF-κB signaling pathway.

## 4. Discussion

IBD is an immune-mediated gastrointestinal disorder marked by persistent mucosal inflammation, primarily driven by an imbalance in pro-inflammatory cytokine production and impaired IEB integrity [21]. The restoration of IEB integrity is thus a promising therapeutic approach for IBD. Our study revealed that BA effectively mitigated IBD pathology by attenuating disease-related weight loss and colonic epithelial injury in a DSS-induced colitis mouse model. Furthermore, in in vitro models (Caco-2/HT29-MTX-E12 monolayers and intestinal organoids), BA enhanced PPAR-γ expression while suppressing inflammatory responses mediated by NF-κB and the MLCK/MLC2 pathway. These actions collectively restored tight junction integrity, positioning BA as a promising candidate for IBD treatment.

To investigate BA’s effects on IEB function in vitro, we used physiologically relevant models that replicate the critical interactions between IECs and immune cells—key drivers of IBD pathogenesis [22]. In IBD, macrophages undergo functional polarization toward a pro-inflammatory phenotype, resulting in the production of inflammatory mediators such as IL-6, IL-1β, and TNF-α, which perpetuate mucosal inflammation. These activated macrophages also hinder epithelial repair mechanisms and compromise tight junctions, exacerbating barrier dysfunction [23]. To simulate the immune–epithelial crosstalk, we co-cultured Caco-2/HT29-MTX-E12 monolayers and mouse intestinal organoids with RAW264.7 macrophages. Upon LPS challenge, we observed a characteristic breakdown of the barrier, evident by reduced TEER and increased paracellular FITC-dextran flux (*p* < 0.01). Notably, BA treatment reversed these effects, normalizing both TEER values and permeability (*p* < 0.05 vs. LPS group), demonstrating its protective effects against LPS-induced barrier disruption.

Chronic inflammation is a hallmark of IBD progression, where the cytokine network becomes dysregulated, leading to a persistent inflammatory state and the loss of mucosal homeostasis [24]. Manar et al. found that BA significantly reduced inflammatory exudation and leukocyte aggregation in an LPS-induced mouse pleurisy model [25]. In addition, studies have shown that BA can also promote NO and TNF generation in macrophages, reduce NF-κB activity, and increase IL-10 production [26]. In line with recent studies, our results demonstrate that LPS-activated macrophages undergo robust pro-inflammatory polarization, releasing TNF-α, IL-6, and IL-1β, which activate key inflammatory pathways, including NF-κB. This results in the upregulation of inflammatory mediators, forming a self-perpetuating cycle of inflammation and barrier dysfunction [27,28]. BA, as a pleiotropic modulator of inflammation, was found to significantly increase the expression of IL-10, a potent anti-inflammatory cytokine, while inhibiting the expression of IL-6, IL-1β, and TNF-α, indicating its potential to alleviate intestinal barrier damage by suppressing pro-inflammatory factors.

Tight junction (TJ) proteins are essential structural and functional components of the IEB. These proteins, such as CLDN1, OCLN, and ZO-1, form a selective paracellular barrier that regulates solute permeability and maintains mucosal integrity [29]. The CLDN1/OCLN complex, anchored to the actin cytoskeleton via ZO-1, is critical for maintaining the physical and functional integrity of the intestinal barrier [30]. The disruption of the TJ architecture and the subsequent mucosal hyperpermeability are early events in IBD pathogenesis. Notably, ZO-1 plays a crucial role in intestinal epithelial renewal, and its deficiency impairs mucosal repair mechanisms [31]. The therapeutic upregulation of TJ proteins strengthens the colonic mucosal defense and may reduce relapse rates by preventing pathogen invasion and immune activation [32]. Luo et al. found that BA inhibits inflammation and mucosal barrier dysfunction, thereby improving T-2 toxin-induced intestinal injury in mice [33]. Our study found that BA effectively increased the expression of ZO-1, CLDN1, and OCLN in Caco-2/HT29-MTX-E12 monolayers and intestinal organoids, suggesting that it may promote the healing of mucosal injuries by enhancing TJ protein levels.

An integrated network pharmacology analysis identified the key pathways through which BA exerts its therapeutic effects in IBD. A KEGG pathway enrichment analysis suggested that BA’s protective effects on IEB dysfunction are likely mediated by the modulation of the PPAR signaling pathway. Peroxisome-proliferator-activated receptors (PPARs) are a family of nuclear hormone receptors that regulate diverse metabolic and immune processes [34]. Among the three subtypes (PPAR-α, PPAR-β, and PPAR-γ), PPAR-γ plays a central role in regulating anti-inflammatory and immune modulatory functions, particularly in IECs [35]. Studies have shown that PPAR-γ is upregulated in colitis models, and clinical data suggest reduced PPAR-γ expression in ulcerative colitis (UC) patients compared to in healthy controls, making PPAR-γ an attractive therapeutic target for restoring intestinal homeostasis [36,37]. Synthetic PPAR-γ ligands, such as thiazolidinediones, exhibit anti-inflammatory effects in IBD models by suppressing pro-inflammatory cytokines and preserving barrier integrity [38]. Studies have found that BA inhibits IL-1β-induced inflammation by activating PPAR-γ in human osteoarthritis chondrocytes [39]. Similarly, our study revealed a strong binding affinity between BA and PPAR-γ. Using the PPAR-γ inhibitor GW9662, we demonstrated that BA’s effects on IEB function were attenuated, suggesting that BA’s protective effects are mediated by PPAR-γ activation.

PPAR-γ activation exerts a multi-tiered protective effect on the IEB by inhibiting IKK-α phosphorylation, which prevents NF-κB p65/p50 nuclear translocation and MLCK expression, thus preserving tight junction integrity during inflammation [40]. PPAR-γ downregulates NF-κB activity by directly binding to the NF-κB p50/p65 dimer, promoting its degradation and inhibiting its translocation into the nucleus [41]. Consistent with this, BA treatment in Caco-2/HT29-MTX-E12 monolayers prevented LPS-induced increases in pro-inflammatory cytokines, such as TNF-α, IL-1β, and IL-6, while maintaining intestinal barrier integrity. Additionally, MLCK activation, triggered by NF-κB translocation, disrupted tight junction proteins such as CLDN1, ZO-1, and OCLN [42]. BA treatment reversed the phosphorylation of NF-κB p65, downregulated MLCK expression, and suppressed MLC2 phosphorylation, further supporting the idea that BA’s protective effects on IEB function are mediated by PPAR-γ activation.

While our study provides valuable insights into BA’s therapeutic potential in IBD, some limitations remain. First, we focused primarily on BA’s effects on intestinal epithelial cells and tight junction proteins but did not address its regulatory role in macrophages. T cells and dendritic cells also play pivotal roles in IBD pathogenesis, and future studies should explore BA’s effects on these immune cells. Additionally, while the PPAR/NF-κB signaling pathway plays a critical role in IBD, it is involved in multiple biological processes such as oxidative stress and autophagy, which should be investigated in future research. A more comprehensive study of BA’s mechanisms across multiple levels will provide a solid foundation for its clinical application in IBD treatment.

## 5. Conclusions

**Figure 7:**
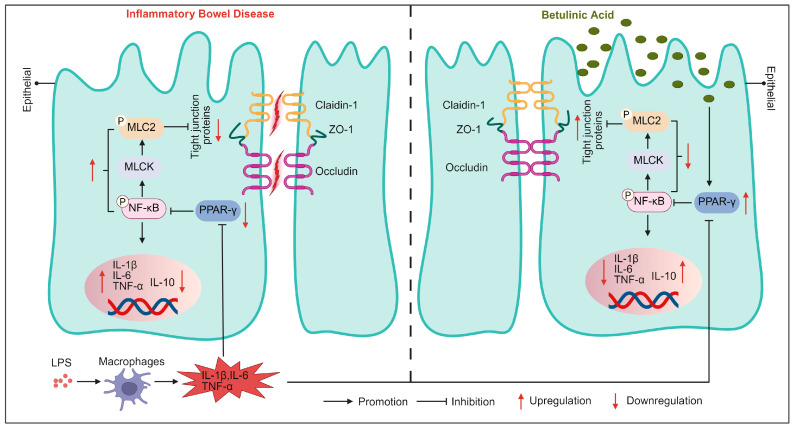
Potential mechanism of BA’s protective role against LPS-activated macrophage-induced disruption of IEB. BA regulates PPAR-γ levels, inhibits NF-κB activation and MLCK expression, suppresses MLC2 phosphorylation, reduces inflammation, and enhances tight junction expression. Created in https://BioRender.com.

In summary, this study established two in vitro models to explore the role of BA in the treatment of IBD from the perspective of the intestinal barrier. BA regulates PPAR-γ levels, inhibits NF-κB activation and MLCK expression, suppresses MLC2 phosphorylation, reduces inflammation, and enhances tight junction expression, thereby treating IBD. This may contribute to the development of new therapies for IBD (Figure 7).

## Data Availability

The original contributions presented in this study are included in the article/Supplementary Material. Further inquiries can be directed to the corresponding author.

## Supplementary Materials

The following supporting information can be downloaded at https://www.mdpi.com/article/10.3390/nu17132052/s1. Table S1: Sequences of the human and mouse gene-specific primers required for qPCR reaction.
