# Supplementary material for: Betulinic Acid Reduces Intestinal Inflammation and Enhances Intestinal Tight Junctions by Modulating the PPAR-γ/NF-κB Signaling Pathway in Intestinal Cells and Organoids
Source: Nutrients. 2025 Jun 20;17(13):2052. doi: 10.3390/nu17132052 (PMC12251175; doi:10.3390/nu17132052)
Supplement: Supplementary file 1 [file nutrients-17-02052-s001.zip › nutrients-3686154-supplementary.pdf]

Betulinic Acid Reduces Intestinal Inflammation and Enhances Intestinal Tight Junctions by Modulating the PPAR-γ/NF-κB Signaling Pathway in Intestinal Cells and Organoids

Xu Zheng [1], Zhen Cao [1], Mingqi Wang [1], Ruqiang Yuan [1,2], Yinhe Han [1], Ang Li [1] and Xiuli Wang [1,*]

[1] College of Basic Medical Science, Dalian Medical University, Dalian 116044, China; zhengxu@dmu.edu.cn (X.Z.); caozhen@stu.glmc.edu.cn (Z.C.); wangmq@dmu.edu.cn (M.W.); yuanrq666@163.com (R.Y.); han1993yh@163.com (Y.H.); drang33@outlook.com (A.L.)

[2] Advanced Institute for Medical Sciences, Dalian Medical University, Dalian 116044, China

* Correspondence: wangxl01@dmu.edu.cn

Supplementary Materials

Table S1. The sequences of the human and mouse gene-specific primers required for qPCR reaction.

Genes	Primers
Human-GAPDH-F	5'-GCTTCCGTGGACACATAAC-3'
Human-GAPDH-R	5'-TGGTGCCTTCTCCCTTC-3'
Human-IL-6-F	5'-GTAGTGAGGAACAAGCCAGAG-3'
Human-IL-6-R	5'-TACATTTGCCGAAGAGCC-3'
Human-IL-1β-F	5'-GGCATCCAGCTACGAATCTC-3'
Human-IL-1β-R	5'-GAACCAGCATCTTCCTCAGC-3'
Human-TNF-α-F	5'-AGCCCATGTTGTAGCAAAC-3'
Human-TNF-α-R	5'-TGAGGTACAGGCCCTCTGA-3'
Human-IL-10-F	5'-TCTCCGAGATGCCTTCAGCAGA-3'
Human-IL-10-R	5'-TCAGACAAGGCTTGGCAACCCA-3'
Human-ZO-1-F	5'-GGTGAAGTGAAGACAATG-3'
Human-ZO-1-R	5'-GGTAATATGGTGAAGTTAGAG-3'
Human-Claudin-1-F	5'-GCTGTGGATGTCCTGCGTGTC-3'
Human-Claudin-1-R	5'-GAGGATGCCAACCACCATCAAGG-3'

Human-Occludin-F	5’-GAGTTGTATCTGTTGTTGT-3’
Human-Occludin-R	5’-TTCGTGGTATAGCATTCT-3’
Mouse-GAPDH-F	5’-TGGCCTTCCGTGTTCCTAC-3’
Mouse-GAPDH-R	5’-GAGTTGCTGTTGAAGTCGCA-3’
Mouse-IL-6-F	5’-CGGCCTTCCCTACTTCACAA-3’
Mouse-IL-6-R	5’-GCCATTGCACAACTCTTTTCTCA-3’
Mouse-IL-1β-F	5’-TGCCACCTTTTGACAGTGATG-3’
Mouse-IL-1β-R	5’-ATGTGCTGCTGCGAGATTTG-3’
Mouse-TNF-α-F	5’-GCCTCTTCTCATTCCTGCTT-3’
Mouse-TNF-α-R	5’-TGGGAACTTCTCATCCCTTTG-3’
Mouse-IL-10-F	5’-CGGGAAGACAATAACTGCACCC-3’
Mouse-IL-10-R	5’-CGGTTAGCAGTATGTTGTCCAGC-3’
